# Community-based trial of annual versus biannual single-dose ivermectin plus albendazole against *Wuchereria bancrofti* infection in human and mosquito populations: study protocol for a cluster randomised controlled trial

**DOI:** 10.1186/s13063-017-2196-9

**Published:** 2017-10-02

**Authors:** Dziedzom K. de Souza, Collins S. Ahorlu, Susan Adu-Amankwah, Joseph Otchere, Sedzro K. Mensah, Irene A. Larbi, George E. Mensah, Nana-Kwadwo Biritwum, Daniel A. Boakye

**Affiliations:** 1 Department of Parasitology, Noguchi Memorial Institute for Medical Research, University of Ghana, Legon-Accra, Ghana; 2 Department of Epidemiology, Noguchi Memorial Institute for Medical Research, University of Ghana, Legon-Accra, Ghana; 3 Neglected Tropical Diseases Programme, Ghana Health Service, Accra, Ghana

**Keywords:** Lymphatic filariasis, Biannual treatment, Twice-yearly treatment, Increased treatment frequency

## Abstract

**Background:**

The Global Programme for the Elimination of Lymphatic Filariasis (GPELF) has been in operation since the year 2000, with the aim of eliminating the disease by the year 2020, following five to six rounds of effective annual mass drug administration (MDA). The treatment regimen is ivermectin (IVM) in combination with diethylcarbamazine (DEC) or albendazole (ALB). In Ghana, MDA has been undertaken since 2001. While the disease has been eliminated in many areas, transmission has persisted in some implementation units that had experienced 15 or more rounds of MDA. Thus, new intervention strategies could eliminate residual infection in areas of persistent transmission and speed up the lymphatic filariasis (LF)-elimination process. This study, therefore, seeks to test the hypothesis that biannual treatment of LF-endemic communities will accelerate the interruption of LF in areas of persistent transmission.

**Methods:**

A cluster randomised trial will be implemented in LF-endemic communities in Ghana. The interventions will be yearly or twice-yearly MDA delivered to entire endemic communities. Allocation to study group will be by clusters identified using the prevalence of LF. Clusters will be randomised to one of two groups: receiving either (1) annual treatment with IVM + ALB or (2) annual MDA with IVM + ALB, followed by an additional MDA 6 months later. The primary outcome measure is the prevalence of LF infection, assessed by four cross-sectional surveys. Entomological assessments will also be undertaken to evaluate the transmission intensity of the disease in the study clusters. Costs and cost-effectiveness will be evaluated. Among a random subsample of participants, microfilaria prevalence will be assessed longitudinally. A nested process evaluation, using semi-structured interviews, focus group discussions and a stakeholder analysis, will investigate the community acceptability, feasibility and scale-up of each delivery system.

**Discussion:**

It is expected that this study will add to the existing evidence on the need for alternative intervention strategies for the elimination of LF in Ghana and in other African countries that are facing similar challenges or are at the beginning of their LF-elimination programmes.

**Trial registration:**

ClinicalTrials.gov, ID: NCT03036059. Registered on 26 January 2017.

Pan African Clinical Trials Registry, ID: PACTR201702002012425. Registered on 23 February 2017.

**Electronic supplementary material:**

The online version of this article (doi:10.1186/s13063-017-2196-9) contains supplementary material, which is available to authorized users.

## Background

Lymphatic filariasis (LF) is a debilitating, mosquito-borne, nematode infection that has been targeted for elimination as a public health problem by 2020. It affects 120 million people in 73 countries where 1.46 billion people are at risk of acquiring the infection through infectious mosquito bites. In 2000, the World Health Organisation, in collaboration with pharmaceutical companies and endemic country governments, launched the Global Programme to Eliminate LF (GPELF). Ivermectin (IVM) in combination with diethylcarbamazine (DEC) or albendazole (ALB) remain the drugs available for the large-scale control of LF [1]. These drugs only temporarily clear microfilariae (mf) without killing all adult worms [2], and it is assumed that a reduction in population mf load will lead to a simultaneous reduction, or even interruption, of transmission [3–5]. It is, therefore estimated, that five to six rounds of mass drug administration (MDA) are required to eliminate the disease. From 2000 to 2014, LF MDA included a cumulative total of 5.62 billion treatments delivered to more than one billion people at least once [6, 7]. Many countries including Ghana have since undertaken between 8 and 15 consecutive annual rounds of MDA in several implementation units without interrupting transmission. In Ghana, where the MDA coverage rates have been more than the required 65%, 29 sentinel sites with persistent residual infections have been identified, with microfilaria prevalence rates of above 1% [8]. Previous studies [9] revealed high prevalence of LF (13% antigen prevalence and 4.6% mf prevalence) in two communities in a district with persistent LF transmission. In these communities, individuals positive for LF were treated and followed at 3-month intervals for 1 year. The results showed a decline in prevalence after treatment, followed by an increase 6 months after treatment (Table 1).

**Table 1:** Number of positives and parasite loads at different time points (Unpublished data – de Souza)

	Dec 2014 (pre treatment)	3 months post treatment	6 months Post treatment	9 months Post treatment	12 months Post treatment
ICT positives	48	17/48 (35.4%)	26/48 (54.2%)	23/33 (69.7%)	15/30 (50%)
mf positives	17/48 (35.4%)	4/48 (8.3%)	4/48 (8.3%)	3/33 (9.1%)	2/30 (6.7%)

*ICT i*mmunochromatographic test, *mf* microfilariae

With the failure to interrupt the transmission of LF in some areas following the recommended years of MDA, alternative and effective MDA regimens and strategies are needed if the GPELF is to achieve the goals of global elimination. Simonsen and colleagues, therefore, recommended treatment to be given at shorter intervals – perhaps every 6 months [2]. Further, a study reported that in villages which were hyperendemic for onchocerciasis, after some 14 years of biannual treatment with IVM, no *Wuchereria bancrofti* could be detected, while in adjacent villages a prevalence of around 3% was found [10]. The elimination of onchocerciasis in some countries in the Americas has been attributed to the use of twice-yearly treatment regimens [11, 12]. Even though there are no studies that explicitly compare the effects of once yearly versus twice-yearly treatment in onchocerciasis control [13], this provides some evidence that biannual treatment may be effective in the control of LF in areas with persistent transmission. In Ghana, the onchocerciasis programme undertakes twice-yearly treatment with IVM in onchocerciasis (river blindness) hyper-endemic districts and it is believed that the use of twice-yearly treatment in these onchocerciasis endemic districts may have contributed to the absence of LF in these districts. However, there is the absence of data to ascertain these observations, since onchocerciasis treatment started in 1996, 4 years prior to the mapping of LF prevalence in the country. Other drugs, such as DEC, have also been shown to have marked effects on mf reduction [3, 14, 15]. However, research to test alternative treatment regimens (including single, high doses of ALB or biannual treatment schedules) have been recommended [1], and concrete evidence is required to inform policy and the need for alternative strategies.

Reviewing the literature using PubMed, Web of Science, Google and other online journals, revealed that there is a lack of information on the use of the twice-yearly treatment strategy for the control of LF. A number of studies have proposed the need for twice-yearly treatment for the control of LF [2, 8, 16]. From these studies, it is clear that increasing the MDA frequency is a considerable global research priority. Two randomised control trials in Mali [17] and Malawi [18] that assessed the dosage and frequency of treatment for LF control have been identified, and have shown the usefulness of increased dosage and frequency of IVM and ALB in suppressing *W. bancrofti* microfilaraemia levels in populations. These, together with the evidence from onchocerciasis studies [10] and anecdotal reports indicate the benefits of the increased treatment frequency. These studies however have limitations in the number of participants randomized and focused on LF control in the general endemic population, compared to the current study in areas with persistent transmission, that face the additional challenge of potential drug resistance by *W. bancrofti*, as a result of the prolonged period of treatment. The impacts of the interventions have also not been assessed in the vectors, especially when different vector species exhibit different transmission processes. This current study will, therefore, assess the impact, feasibility and acceptability of the twice-yearly treatment for the control of LF in both human and vector populations, and will be useful in the implementation of the twice-yearly treatment in countries in Africa that are yet to start MDA or are at the early stages of their implementation programmes.

To advance knowledge for the control of LF in areas with persistent transmission, the trial proposed herein will address the following questions: (1) Can a twice-yearly treatment with IVM and ALB eliminate residual LF infections in areas with persistent transmission? and (2) Can the implementation of the twice-yearly treatment accelerate the interruption of transmission of the disease in these areas? This study will test the hypothesis that biannual treatment of LF-endemic communities will accelerate interruption of LF in areas of persistent transmission in Ghana.

### Study aims and objectives

The primary objective of this trial is to interrupt the transmission of LF with a twice-yearly treatment with IVM and ALB. The secondary objectives are to assess the impact of the twice-yearly treatment on transmission of parasite in areas of persistent transmission and evaluate the cost-effectiveness of a twice-yearly treatment versus the current yearly treatment based on IVM and ALB. The study will also include process and economic evaluations to assess the feasibility and implementation of the twice-yearly treatment, which will guide scale-up similar interventions in other settings in Africa. The more detailed study objectives are:

1. To quantify the impact of twice-yearly versus yearly community treatment (treatment strategies and delivery systems) in reducing parasitological and entomological LF transmission indices in ‘hotspot’ areas
2. To evaluate the costs and cost-effectiveness of twice-yearly treatment in reducing LF transmission
3. To assess the extent to which the twice-yearly treatment is acceptable to the community and feasible, given the health system capacity, and can be easily scaled-up elsewhere
4. To assess the impact of IVM and ALB treatment on other intestinal and soil-transmitted helminthes (STH)

### Study design

This is a phase-4 study of an approved drug, with the aim to delineate additional information for its optimal usage and efficacy. It is designed as a cluster randomised, open-label trial with two study arms, with the ultimate aim of comparing the endpoint results in the intervention versus control arm. A community cluster randomised trial in different settings in Ghana will evaluate the impact and cost-effectiveness of twice-yearly and yearly MDA. The primary outcome is the prevalence of LF. This outcome is selected because it is the prevalence of LF that determines the level of on-going transmission (both parasitological and entomological) and the need to implement Transmission Assessment Surveys and stop MDA [19]. Allocation to study group will be by villages, identified through programme reports, and by disease-control officers in the study districts. The mf prevalence in all communities will be re-evaluated, based on nucleopore filtration of 1 ml of night-blood collected from study participants, prior to assignment to study groups.

The two study groups are:

1. Control group: annual MDA. Clusters (with members aged 5 years and above) will receive 400 μg/kg IVM + 400 mg ALB from trained community drug distributors (CDDs), as part of the ongoing national MDA programme
2. Expanded frequency group: annual MDA with 400 μg/kg IVM + 400 mg ALB, followed by an additional MDA 6 months later. The drugs will be provided by the national programme and administered by the CDDs, following the structures of the National MDA programme

### Study outcomes and measurements

**Fig. 1:**
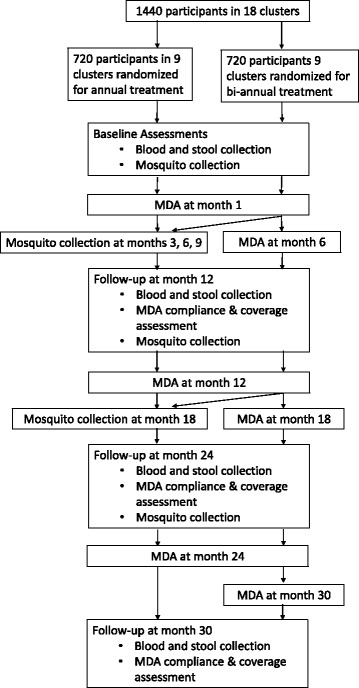
Flowchart of study activities

The primary outcome, the prevalence of LF infection, will be measured through cross-sectional parasitological surveys conducted at baseline and at 12, 24 and 30 months’ follow-ups. Figure 1 presents a flowchart of the study design. The timing of the final follow-up survey will take into account differences in time since treatment of the annual and biannual treatment groups at 24 months. The time point assessments at 12, 24 and 30 months will allow us to evaluate changes in the primary study outcome in the intervention and control groups over time. For secondary outcomes, a subsample of individuals from the clusters in each of the study groups will be followed longitudinally for 2.5 years in order to better understand the transmission dynamics of LF and to estimate key parameters for mathematical modelling of transmission dynamics and treatment impact. The impact of treatment on other STHs (roundworm (*Ascaris lumbricoides*), whipworm (*Trichuris trichiura)*, and the hookworms (*Ancylostoma duodenale* and *Necator americanus*) and any other species identified) will also be assessed. A nested process evaluation, using semi-structured interviews, focus group discussions (FGDs) and a stakeholder analysis, will investigate the community acceptability, feasibility and participation in the twice-yearly drug administration. The local and regional health system structures and processes will be evaluated to determine the feasibility to scale-up the interventions.

## Methods

**Fig. 2:**
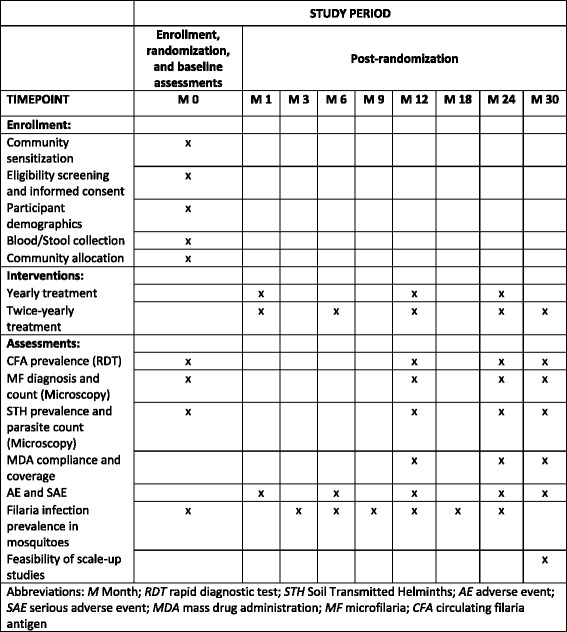
Standard Protocol Items for Randomised Trials (SPIRIT) Figure – Schedule of enrolments, interventions and assessments

The publication of this research protocol follows the SPIRIT (Standard Protocol Items for Randomised Trials) recommendations [20]. For the SPIRIT Checklist see Additional file 1, and for the SPIRIT Figure see Fig. 2. Table 2 provides an overview of the trial characteristics.

**Table 2:** Trial registration data

Data category	Information
Primary registry and trial identification number	ClinicalTrials.gov, ID: NCT03036059
Date of registration in primary registry	26 January 2017
Secondary identifying numbers	Ghana Health Service Ethics Review Committee: 04112/2016 Noguchi Memorial Institute for Medical Research IRB: CPN 062/16-17
Source(s) of monetary or material support	EDCTP grant TMA 2015 CDF - 976
Primary sponsor	Noguchi Memorial Institute for Medical Research
Contact for public queries	DKdS
Contact for scientific queries	DKdS, CSA, SAA
Public title	Twice Yearly Treatment for the Control of LF
Scientific title	Cluster Randomised Community-based Trial of Annual Versus Biannual Single-dose Ivermectin Plus Albendazole Against Wuchereria Bancrofti Infection in Human and Mosquito Populations
Country of recruitment	Ghana
Health condition(s) or problem(s) studied	Lymphatic filariasis
Interventions	400 μg/kg ivermectin + 400 mg albendazole tablets given once or twice a year
Key inclusion and exclusion criteria	Inclusion criteria: residency in endemic community for at least 12 months; willingness to provide informed consent/assent; willingness to donate blood (per the protocol) Exclusion criteria: recent residents (<12 months) in the study districts; inability to give informed consent due to illness, serious medical problems or refusal to participate in the study; pregnancy; children below the age of 5 years
Study type	Interventional allocation: randomised Masking: no masking, open label
Date of first enrolment	19 May 2017
Target sample size	1440
Recruitment status	Recruiting
Primary outcome(s)	Change from baseline prevalence of lymphatic filariasis at 12, 24 and 30 months
Key secondary outcome(s)	Longitudinal assessment of transmission dynamics of lymphatic filariasis. Evaluation of community acceptability of twice-yearly treatment. Feasibility of scale-up of twice-yearly treatment

### Study population

This study will be undertaken in the Western Region of Ghana, which lies within the high rain forest vegetation climate zone of the West African subregion, with strands of mangroves. The highest mean temperature is 34 °C while the lowest is 20 °C. Relative humidity is very high, averaging between 75% to 85% in the rainy, and 70% to 80% in the dry seasons. Rainfall is experienced throughout the year with the highest monthly mean occurring around May and June (http://mofa.gov.gh/site/?page_id=1778). The main economic activities in the region are agriculture and fishing. The region has a commercial capital, Takoradi and an administrative capital, Sekondi, located about a 3.5-h drive away from Accra, the capital of Ghana. Besides the main administrative capitals, the communities in the districts are predominantly rural. The region is one of the highest endemic zones in the country [21].

The sites selected are areas that have had 13–15 rounds of yearly treatment, without interrupting transmission of LF. The study areas selected are also among the highest endemic communities (zones) in the country. Undertaking the study in these sites, therefore, provides the opportunity to assess the impacts of the intervention.

**Fig. 3:**
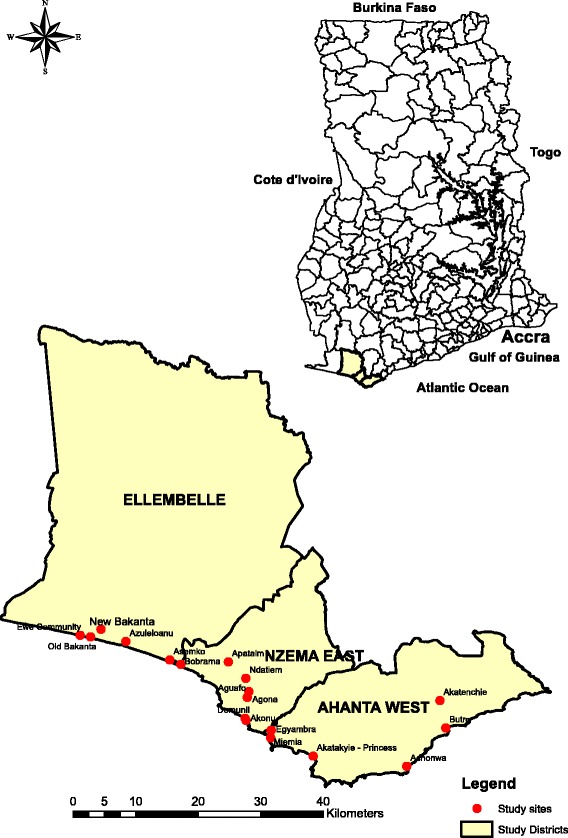
Study districts and communities

The study will be carried out in 18 clusters in the Western region of Ghana. Communities were selected following a review of the Neglected Tropical Disease Programme (NTDP) sentinel and spot check site monitoring data, as well as recommendation from the District Health Management Team (DHMT). Based on these, 18 communities were selected, distributed into three districts in the Western Region of Ghana (Fig. 3). In these districts, the persistent transmission of LF has been reported following several rounds of MDA [8] and previous studies also indicated inaccuracies in the treatment coverage data reported [22].

### Sensitisation and recruitment

Key stakeholders will be involved in the study and its design. Prior to implementation, meetings will be held with the NTDP, and at each study site, where key stakeholders (chiefs, community leaders and study participants) will be sensitised about the study objectives, interventions, evaluation procedures, and requested to provide input as well as the need for their participation in the study. Community meetings will be held to describe the purpose of the study, the interventions, the evaluation procedures to be followed, and the risks and benefits associated with participation. Individuals will have the opportunity to ask questions for which answers will be provided by the research team.

Consent for the intervention will be provided at the community level with the option for individuals to opt out. Community drug distributors (CDDs) used by the NTDP will number all houses and update their community registers through coordination with the chiefs and village elders. Using a random number generator in Excel, households will be selected until the sample size for each village is attained. Subsequently, households selected for inclusion into the study will be visited by field staff, for consenting and recruitment into the study. In each household visited, written informed consent to conduct the household-level questionnaire will be sought from the household head. Individual-level consent will be sought from selected individuals, 18 years old and above (either for themselves or their children) and written assent sought from children aged 12–17 years.

### Survey procedures

At each house, household heads will be interviewed to collect information on household characteristics and other information during household visits. Data on sex, age, occupation, participation in previous MDAs and duration of residence will be recorded. Information on mosquito nets or other barriers present in the house will also be recorded.

CDDs will update their treatment registers with a full record of all individuals who have received treatment. To augment these data, population-based coverage surveys, using multistage clustering sampling [23], will be carried out among a random subsample of communities. In each cross-sectional survey, 100-μl finger-prick blood samples will be collected (from selected individuals) during the day and tested for circulating filarial antigen (CFA) using the Alere Filariasis Test Strips (FTS) [24]. Individuals positive for LF antigen will be followed for night-blood collection, for mf detection [25]. To enhance the sensitivity of microfilaria detection, the nucleopore filtration method using 1 ml of blood will be employed. Two-milliliter venous blood samples will be collected in heparinised capillary tubes at night between 9 p.m. and 2 a.m., and used in the nucleopore filtration method. The night collection will be done between these periods because *W. bancrofti* shows nocturnal micofilariae level periodicity, which peaks within 9 p.m. to 2 a.m. Arrangements will be made with the study participants on the timing for the blood collection. Ten percent of all samples will be evaluated for quality control by a supervisor.

IgG4 antibody testing using the Wb123 assay will be used in a subset of study participants (individuals positive for LF antigen and children aged 5–10 years), and the results evaluated together with the antigen and microscopy results. The evaluation of the test in individuals positive for LF antigen is done to identify new from existing infection, while the selection of children 5–10 years is done to assess the usefulness of the antibody testing for Transmission Assessment Surveys, used for determining the criteria for stopping MDA [19]. However, while the presence of IgG4 may reflect exposure to infective parasite (antibody responses develop before patent infection and, as such, positive responses may be suggestive of recent filarial exposure), antibodies take a long time (sometimes years) to normalise after treatment [26], and detection of antibody response in populations that have been treated only shows that these have been exposed at some point. Pilot assessments in a limited population of individuals treated with IVM and ALB pointed to implementation and interpretation challenges [27]. The inclusion of the Wb123 assay in the study thus provides an opportunity to further evaluate its usefulness when undertaking impact/evaluation assessments. As such, the usefulness of this test on the overall outcome will be considered carefully in the data analysis.

In addition, samples will be stored (for up to 10 years) for future studies at the Noguchi Memorial Institute for Medical Research (NMIMR), including the detection of potential drug-resistance alleles and genome sequence analysis to investigate the genetic structure of *W. bancrofti* populations. All members of the study teams will be appropriately trained in the study objectives and procedures. Standard operating procedures (SOPs) will be developed, used to guide all field and laboratory activities. Supervisors will make regular visits to the field to monitor the activities.

Future use of the samples for purposes not specified in the Information Document for Participants will have to be approved by the Ethics Committee/Review Boards in Ghana which approved this study. The use of these samples by external parties to the current study (whether for research specified in the Information Document or beyond) will be based on a Material Transfer Agreement.

### Longitudinal surveys

Individuals who are positive for LF antigen and microfilaria (at baseline) will be followed longitudinally to help quantify the transmission dynamics of LF. They will be asked to provide blood samples 1 month following treatment. Two milliliters of venous blood will be collected in heparinised capillary tubes at night between 9 p.m. and 2 a.m. The collected samples will be transported to the laboratory and analysed. Subsequently, they will be revisited at 3, 6, 9 and 12 months post treatment and asked to provide a sample, which will be examined for the presence of *W. bancrofti* mf [21, 22]. They will also be requested to provide stool samples for the examination of STH infections.

The study team will also conduct household visits to assess the extent of non-compliance to treatment and factors associated with non-compliance [28–33]. The assessment of non-compliance will be based on short, standardised questions, as well as a review of the community drug distribution register.

### Sample size calculation

The sample size was calculated based on the null and alternative hypotheses, together with the type-I and type-II errors defined as follows:

Null hypothesis (H_0_): the additional dose of IVM is not more effective than the standard single dose per annum treatment (i.e. Treatment – Control = 0).

Alternative hypothesis (H_A_): the additional dose of IVM is more effective than the standard dose (i.e. Treatment − Control > 0).

The type-1 error rate (*α*), i.e. rejecting H_0_ when it is true, is set at 0.05, with a two-sided test. To control for type-II error (*β*), the probability of rejecting H_0_ when it is false is set at 0.80, taking into consideration budget allocation.

The hotspot districts have been receiving an annual single dose of IVM for the past 15 years with the prevalence rates ranging between 1% and 18.2%. Therefore, it is assumed that the new intervention would have an effect, expected to be larger than the effect of the standard control approach, with an effect size of 0.4. Based on these assumptions, the sample size was calculated using the Optimal Design software [34]. Thus, for 0.80 power the study requires a cluster size of 58.

With an expected non-response and a loss to follow-up of 37% (determined from previous unpublished studies), the sample size per cluster has been determined to be 80. With 18 clusters to be surveyed, a total of 1440 study participants, from the two study districts, will be expected to take part in this trial.

Study participants will not be paid as part of the survey. However, in order to mitigate the high dropout rates during follow-ups, participants will be compensated for the time lost for working during the days they are participating in survey activities, with an amount of 7 Cedis (equivalent to US$1.61). They will also be contacted at 14 days and 7 days prior to follow-up, in order to ensure their availability. Even though there is always the possibility of emergencies and other social responsibilities that may result in their unavailability, efforts will be made to reach them.

### Treatment interventions

All study groups will receive treatment with 400 μg/kg IVM + 400 mg ALB, which is highly efficacious against *W. bancrofti* [1]. The difference between the study groups is the frequency of treatment provided (Table 3). The MDA will be implemented in all communities as part of the ongoing NTDP. In Ghana, MDA started in the year 2000 as part of the GPELF. Treatment with 400 μg/kg IVM + 400 mg ALB is provided annually by trained CDDs. Every year, the national team organises training for regional, district, subdistrict supervisors, CDDs and their supervisors. They are also taken through their roles and responsibilities. The programme targets all eligible individuals aged 5 years and above in all endemic districts and communities. The strategies follow the door-to-door Directly Observed Treatment (DOT) strategy [35]. This study will follow the exact MDA strategy used by the NTDP and no modifications other than the frequency of treatment will be made. All treatments will be undertaken through the systems established by the NTDP. Further, the onchocerciasis programme in Ghana undertakes twice-yearly treatment with IVM in hyperendemic areas, and the twice-yearly treatment in LF-endemic areas will be an extension of the onchocerciasis program.

**Table 3:** Drug dosage and schedule

Arms	Assigned interventions
Control group	400 μg/kg ivermectin + 400 mg albendazole tablets given every year for 2 years
Expanded frequency group	400 μg/kg ivermectin + 400 mg albendazole tablets given every 6 months for 2 years

Routinely, treatment by the NTDP is done from house to house. CDDs also visit schools to treat after prior notification. Treatment is also done at the markets and sometimes at the church and mosque. Work in the districts is monitored and supervised by coordinators. The team supervisors supervise five or six teams, each of which is made up of two CDDs. Communities are demarcated into sections and marked according to days they are to be visited. Teams under a supervisor work in demarked sections in a ‘sweeping’ fashion one after the other each day (i.e. the whole group move to a section, finish the work there and move to the next). The last day is used to ‘mop up’ though all the sections of the area to which the group has been allocated.

### Drug manufacturers and supply

Ivermectin is manufactured by Merck & Co. Ivermectin for the control of elephantiasis and onchocerciasis is donated to national disease elimination programmes for free. At the London Declaration in 2012, the World Health Organisation (WHO) together with pharmaceutical companies and donors committed to: ‘Sustain, expand and extend programmes that ensure the necessary supply of drugs and other interventions to help eradicate Guinea worm disease, and help eliminate by 2020 lymphatic filariasis, leprosy, sleeping sickness (human African trypanosomiasis) and blinding trachoma’. As such, the drugs used by the Ghana Neglected Diseases Programme of the Ghana Health Service, are procured through the systems established by the WHO. As such every year, depending on the target population to be treated, the NTDP formally requests the supply of drugs from the WHO. The WHO facilitates the supply of the IVM and ALB for LF-elimination programmes. A joint mechanism and a set of forms have been developed to facilitate the process of application, review and reporting. The request forms are signed by the NTD programme manager to formally endorse the request for the drugs, and submitted to the WHO country office, with electronic copies to PC_JointForms@who.int and the concerned regional focal point, no later than 15 August of the year preceding the year for which medicines are intended to be used (e.g. at the latest by 15 August 2017 for implementation of preventive chemotherapy in 2018) but at least 6 − 8 months before the planned intervention(s) to allow time for reviewing and approval of the request, placing order, drug manufacturing and shipment to the country. All drug request forms are available at http://www.who.int/neglected_diseases/preventive_chemotherapy/reporting/en/.

The request is submitted to the WHO AFRO for review by the Regional Programme Review Group (RPRG), i.e. a technical review committee convened by the AFRO Regional Office as part of the Joint Application Package (JAP). After the RPRG’s approval the request is submitted to the WHO Headquarters (WHO HQ) in Geneva which proceeds to order the shipment after satisfying themselves regarding the approved request. The WHO HQ is responsible for the procurement process. The drug company, after receiving the request, ships the consignment from door to door, i.e. from the factory to the Central Medical Stores (CMS) in Ghana. Countries need to provide tax exemptions for the shipment before the drugs are shipped to the country. In Ghana, the WHO office facilitates the tax exemption process and receives the consignment of drugs on behalf of the programme and then hands over the consignment to the program.

Once the country takes over the drugs in the CMS, a distribution list for regional distribution and a request are sent to the Head of the CMS who proceeds to distribute the drugs to the Regional Medical Stores which then distribute the drugs to the districts, then to the subdistricts and then eventually to the CDDs who do the house-to-house distribution of drugs during the MDAs. Below the regional level, disease-control officers take charge of the drugs and their distribution to the next level.

### Cluster randomisation

Randomisation will be stratified by the prevalence of LF (as determined in the baseline surveys). The randomisation sequence generation will be undertaken by an independent statistician using computerised random-number generation. Sealed envelopes containing cluster identification will be placed in pre-stratified ballot boxes, with community leaders invited to select envelopes from the boxes and directed to put the selected envelope in a box labelled A or B (corresponding to the two study groups, each with nine clusters) according to the pre-generated randomisation sequence for that stratum. With an average household size of six and the frequency of under 5 years being 14.3%, 45 households will be randomly selected in each cluster and five eligible household members recruited into the cross-sectional surveys. Owing to the nature of the interventions, participants will not be blinded to their group randomisation. However, the identity of the study groups will remain hidden until the completion of community sensitisation and randomisation in order to eliminate participation bias. Further, the laboratory technicians conducting the parasitological examinations and the statistician responsible for analysis will be blinded to the group assignment. The blinding to the laboratory technicians and statistician will be done using sample/study participant identification codes for samples and results which will not be linked to the study groups.

### Entomologic surveillance

Surveys for vectors and transmission of LF will be carried out quarterly throughout the first year, and subsequently every 6 months. Vector collection will be carried out in selected villages. To ensure that mosquitoes do not cross over between communities, vector collection communities will be at least 2 km apart.

In each community collections will be undertaken in 10 selected participants’ households, distributed so as to be representative of the community. The community will be divided into four approximate sections. All households in the section will be numbered. The day before the planned collection, the dice will be rolled to select the households. For example, if the dice is rolled and the number is 5, then the fifth house will be selected. Upon selection, the household head will be approached, the study explained and consent sought. If a household refuses participation or household members are absent, a different household will be selected as a replacement. Subsequently, the sum of the dice rolls will be added to the previous number, for the selection of the remaining households. At least two households will be selected per section.

Collections will be undertaken using the Pyrethrum Spray Collection (PSC) in the rooms provided by the consenting individuals. The PSC will be undertaken in the morning from 5 a.m. to 8 a.m. White bed sheets will be laid on the floor and other surfaces in the rooms, after mosquito hiding places (under the bed, tables) have been disturbed to displace any resting mosquitoes. The room will then be sprayed with pyrethrum insecticide (RAID insecticide), commonly found on the Ghanaian market) and left for about 15 min, after which the white sheets will be inspected for any dead or knocked down mosquitoes. The mosquitoes will be collected and placed in a petri-dish labelled with the village name, GPS coordinates and collection date. Collected mosquitoes will be identified using morphological identification keys [36, 37]. All mosquito collections will be undertaken by trained entomologists at the NMIMR.

### Inclusion/exclusion criteria

The NTDP and WHO recommendations for the treatment of endemic communities and monitoring and evaluation of treatment programmes, target all individuals aged 5 years and above. As such, the same population will be used in this study. Only healthy volunteers, both men and women will be recruited into the study. The following inclusion and exclusion criteria will apply in the selection of study participants:

#### Inclusion criteria

Criteria for eligibility to participate in the human study:

- All study participants must be resident in the disease endemic community for at least 12 months
- All study participants must be willing to provide informed consent/assent
- All study participants must be willing to donate blood (per the protocol)
- For mosquito collectors only: volunteers over the age of 18 years who have received formal training in safe and scientifically reliable mosquito collection

#### Exclusion criteria

The following individuals are ineligible to participate in the study:

- Individuals who are recent residents (less than 12 months) in the study districts.
- Individuals who are unable to give informed consent due to illness, serious medical problems or refusal to participate in the study
- Pregnant women – determined through questioning or visual examination at the time of consent
- Children below the age of 5 years

### Data management and analysis

Field and laboratory data will be recorded by technicians using Samsung Galaxy Tabs A7.0., and entered into a customised database. All data collections tools are coded using CSPro and the data will be transferred onto a secured server.

The analysis will be aimed at (1) assessment of any differences in baseline characteristics between treatment groups, (2) intent-to-treat analysis, i.e. assessment of the effectiveness of the single or twice-yearly treatment regimens on infection intensities and disease outcome and (3) assessment of differences in adherence to the treatment regimens between the groups, potential confounding, and adjusted effectiveness analysis.

All statistical analyses will be based on the clusters with both baseline and endpoint parasitological results. Results will be presented as appropriate effects sizes with a measure of precision (95% CIs). Unadjusted and adjusted results will be presented for all analyses. Covariates in adjusted analyses will be predefined and will include participation in previous MDAs and access and use of bed nets. For continuous outcomes, analyses will adjust for baseline by inclusion of the cluster mean of the outcome in question as a covariate in statistical models.

Demographic and population characteristics of clusters will be compared between study groups. These measures will be tabulated, but no significance tests will be performed to investigate for differences between groups at baseline. Statistical testing will be restricted to comparison between the two community-based treatment groups. Secondary outcomes will be pre-specified for statistical testing along with the primary outcome.

The summary statistic for each parasitological endpoint will be estimated, and compared using paired Student’s *t* tests. Changes in rate of infection will be expressed as percentage differences from the pre-treatment rates. The significance in differences in mosquito infection rates will be tested using the chi-square test. The significance of correlation between different indices will also be tested using the chi-square test. The significance of differences in monthly transmission potential before and after treatment will be tested with the non-parametric Mann-Whitney *U* test. Analysis of variance (ANOVA) will also be used in the intent-to-treat analysis for comparing the study groups. SPSS for WINDOWS 16 (SPSS Inc, Chicago, IL, USA) will be used for all statistical computations. The mf intensities will be adjusted for sampling time by multiplying the counts with a time-specific factor [38]. Geometric mean intensities (GMIs) of microfilaraemia and antigenaemia prevalence will be calculated [39].

Below is the statistical analysis strategy.

## Primary outcome: prevalence of LF infection measured at 12, 24 and 30 months post intervention

### Testing the study hypothesis

The additional treatment that will be administered to the intervention group, 6 months after the national mass drug administration (MDA) for 2 consecutive years, will constitute the main intervention in this study. The effect of the intervention will then be assessed by measuring our primary study outcome in both the control and intervention groups at 12, 24 and 30 months. Therefore, our study hypothesis will be tested by comparing the rates of the primary study outcome in the intervention and control groups over time.

#### Pre-post analysis

Since the emphasis is on the comparison of the intervention and control groups, each of the follow-up measurements will be analysed in relation to the baseline measurements of the outcome in a pre-post analysis. For instance, Y_i0_ = 1 to Y_i1_ = 0 in Eq. 1 would indicate a favourable effect of the intervention in group 2 while Y_i0_ = 1 to Y_i1_ = 1 would indicate no effect and Y_i0_ = 0 to Y_i1_ = 0 will be considered a preventive effect:

1 $$ \left\{\begin{array}{l}{Y}_{\mathrm{i}0}=1\to {Y}_{\mathrm{i}1}=0\hfill \\ {}{Y}_{\mathrm{i}0}=0\to {Y}_{\mathrm{i}1}=1\hfill \\ {}{Y}_{\mathrm{i}0}=0\to {Y}_{\mathrm{i}1}=0;\kern1em {Y}_{\mathrm{i}0}=1\to {Y}_{\mathrm{i}1}=1\hfill \end{array}\right., $$

where the baseline measurement of the outcome for the i^th^ participant is given by Y_i0_;

Y_i1_ denotes the measured outcome at the first follow-up; and 1, 0 indicate the presence or absence of LF infections, respectively.

Therefore, by subsetting on each of the possible scenarios of the baseline measurements (Eq. 2), the control and intervention groups could be compared using second-order transition models where separate logistic regression models will be fitted for each of the possible scenarios:

2 $$ \left[\begin{array}{ll}\upchi 00\hfill & \upchi 01\hfill \\ {}\upchi 10\hfill & \upchi 11\hfill \end{array}\right], $$

where χ_00_ is the probability that the follow-up measure of the outcome and Y_ij_, is 0 when the baseline measurement is also 0.

Results will be presented separately for every time point and then after the study, the overall effect of the intervention will be analysed, taking all time points into consideration. Further, after the first time point we would employ a generalised linear mixed model to account for the correlation between the repeated measurements per individual.

### Secondary outcomes

A subsample of 80 individuals from the following clusters: low (1%), medium (2–4%) and high (>5%) LF prevalence; in each of the study groups will be followed longitudinally for 2.5 years in order to better understand the transmission dynamics of LF, and to estimate key parameters for mathematical modelling of transmission dynamics and treatment impact. Both the presence and intensity of *W. bancrofti* mf will be assessed at 3, 6, 9 and 12 months post treatment. Information on covariates that influence *W. bancrofti* mf transmission in humans, such as use of bed nets, will be measured at each of these endpoints.

#### Analysis strategy

Each of our study outcomes, i.e. prevalence and intensity of *W. bancrofti* mf, will be considered separately during the analysis. Loss to follow-up was taken into consideration during our sample size calculation. Therefore, regardless of the number of incomplete observations, we expect to end up with no less than 58 complete observations per community at the end of the study.

A panel of individual line plots for the study participants would be used in the assessment of within-person variability. Since the assumptions of likelihood are violated by the effect of within-group and within-subject clusters in longitudinal data, in assessing the transmission dynamics of LF infection, the measured covariates will be regressed separately on both the prevalence and the intensity of infections using a quasi-likelihood method, i.e. generalised estimating equations, which uses an association matrix instead of the diagonal matrix employed by the generalised linear models. As part of the model validation, the point-referenced residuals of the provisional models will be assessed for spatial autocorrelation. The results of this assessment would help to determine if unmeasured/unobserved factors in the study areas play relevant roles in the transmission dynamics of LF infections. Using classical geostatistical concepts, our final model would be adjusted for both the effects of the measured and unobserved factors.

For entomological endpoints, infection rates will be determined, using the polymerase chain reaction (PCR) [40] and LAMP [41] methods. The results will be analysed using the poolscreening software by Katholi and colleagues [42, 43].

The findings and data from this study will be used to develop a mechanistic model, which describes the variability in treatment responses in populations. This will be implemented into an already available and well-established transmission dynamic model for LF (LYMFASIM) [44, 45], which will then be used to systematically explore the consequence of twice-yearly treatment on the long-term impact of MDA and elimination prospects. LYMFASIM simulates the spread of *W. bancrofti* in a human community and the impact of control measures, with the advantage of accounting for a variety of parameters specific to the population of interest and output predictions [45, 46].

### Process evaluation

A key study objective is to understand the process and structures that support the delivery of twice-yearly MDA to guide its scale-up in Ghana and elsewhere in Africa. Therefore, there will be continuous monitoring of all the implementation processes to determine whether the interventions are being implemented appropriately. The monitoring data will also help to determine what needs to change for the intervention to make an impact on the outcome variables to be measured. This would allow for the documentation of all changes that may occur during the implementation process to aid decisions on the scaling up of interventions. The process evaluation will be done using qualitative data collection techniques to generate data to complement the quantitative data to be generated through baseline, midline and end-line surveys as well as provide contextual information to enhance proper interpretations of study results.

Qualitative evaluation will seek to identify and describe key assumptions and conditions underlying the implementation, sustainability and scaling up of the different strategies and delivery systems. The focus of the evaluation will centre on whether CDDs can be utilised for the effective delivery of chemotherapy for control of LF and what factors influence the use of CDDs, including what type of incentives, if any, should be given. Investigation will focus on: (1) community acceptability, which will be assessed using FGDs and in-depth interviews (IDIs) and (2) feasibility, including a situation and stakeholder analysis of the structural, organisational and management factors that enhance or constrain effective implementation [47]. A series of FGDs will be conducted with community members, CDDs and local health officials, to better understand the acceptance and implementation of the interventions, using predefined and structured topic guides. IDIs will be carried out with a range of actors and opinion leaders in order to understand the process and constraints of the different delivery systems. The number of IDIs will depend on when theoretical saturation is reached, but they will include members of the district, regional and national health teams.

FGD participants will be randomly selected from adults aged 18 years and above in each community. It is hoped that a maximum of four FGDs will be conducted in each community, made up of two each of male and female groups. In communities, where there are six or more drug distributers, they will constitute a discussion group; or else in-depth interviews will be conducted with them. Also, FGDs will be conducted with local health officials from the health facility nearest to the study communities. Again, where the numbers are less than six; in-depth interviews will be conducted with them individually.

In-depth interviews will be conducted with two staff each from the district, regional and national health directorates. The selected individuals will be those who are involved in the LF control/elimination programme in one way or another. The place where FGDs and IDIs will be conducted will be left to the convenience of the participants, but with no, or very minimum, interruption during the discussion session. In-depth interviews will also be conducted at a place convenient to the respondent; however, the privacy of the respondent will be ensured.

FGDs and interviews will be digitally recorded, with notes additionally taken, transcribed and translated. Transcripts will be imported into MAXQDA (VERBI GmbH), coded by two independent coders, and analysed using content analysis to identify emerging themes [48]. Following descriptive analysis, patterns and linkages among views, experiences and behaviours of participants will be explored. The collected data will provide important contextual information and a basis for evaluating the generalisability of the study findings.

### Cost analysis

The cost will be estimated as the sum total of expenditures incurred on each treatment arm. An ingredients approach will be used for the collection of cost data, and by consultation of the LF programme accounting system. Standardised frameworks will be used, capturing fixed and recurrent costs incurred at community levels. For the analysis, the cost will include only expenses directly related to the treatment activities. All costs including both cash and in-kind contributions will be used to estimate financial and economic costs of the alternative treatment strategies (annual versus biannual treatment) and delivery systems (through CDDs). Financial costs will reflect the unit cost of the drug distribution and the resources required for its delivery in terms of the actual expenditures incurred. The economic costs will reflect the opportunity cost of all the resources (financial or in-kind contributions) utilised during the intervention. For example, the time spent by health personnel involved in the intervention will represent an economic cost since the personnel are already receiving a salary and may not receive additional income as part of their involvement in the intervention. The financial and economic costs to be assessed will include the direct financial costs, opportunity costs, advocacy, mobilising the community, training of community volunteers and payments made to community volunteers for drug distribution. Other indirect costs, such as expenses made on the surveillance procedures, will not be included in the analysis. It is also important to note that the actual costs of the drugs will not be included in the analysis since these are donated for free to the NTDP. The determination of capital costs will be based on the useful life of equipment including motorbikes, vehicles and other assets. Capital costs will be annuitised using a discount rate of 3% [49]. The costs will also be classified under provider and society-related costs. The provider costs relate to the costs borne by the public health system in providing the intervention, while the societal costs refer to the wider direct and indirect costs not only to the provider but also to the household in terms of their lost time and income. The cost will be analysed in terms of the overall treatment effectiveness, thus providing information on the cost of the intervention strategy and its effectiveness. The cost will also be analysed based on the volume of treatments distributed, thus enabling the determination of cost functions. The treatment interventions will provide protection against LF for a number of months, and the duration of protection offered will have cost implications as it will determine when the next intervention needs to be delivered. Our preliminary data (Table 1) reveal a rise in mf prevalence 6 months after treatment, and thus providing twice-yearly treatment will provide constant protection to the community. To allow greater comparability between the interventions arms the standardised costs of protecting one person for a full year IVM + ALB will be calculated. An important aspect of the cost analysis in terms of programme implementation will be the scale-up of the biannual treatment in other endemic areas with persistent transmission. As such, itemised costing and sensitivity analysis will be undertaken to enable the estimation of the costs of scaled up implementation.

### Ethics approval and consent to participate

The study (Version 1.2 dated 6 April 2017) was reviewed and approved by the Ethics Committee of the Ghana Health Service (GHS-ERC: 04112/2016). It was also reviewed by the NMIMR IRB (CPN 062/16-17) with Federal Wide Assurance Registration (FWA 00001824). Any substantial changes to the study protocol and other documents will be filed as an amendment with the aforementioned ethics committees. The international standard for the design, conduct, monitoring, and reporting of clinical research of investigational drugs (ICH E6 GCP) will be followed in this study. Compliance with Good Clinical Practice (GCP) standards will enhance the protection of study participants and the integrity of the data collected during the study. In addition, ethical standards and guidelines of Horizon2020 will be adhered to in this study.

Prior to conducting demography and obtaining informed consent, repeated community meetings will be held in all of the villages to communicate the purposes of the study and answer questions at the individual and community level. IVM and ALB, the drugs to be used in this study, are safe and used in the yearly mass treatment for the control of the disease. Even though adverse reactions to the drugs are not common, monitoring of SAEs will be done following the procedures used by the NTDP and all individuals reporting side effects to the drugs will be referred to the nearest hospital and managed appropriately.

All personal information will remain confidential. Laboratory specimens, reports, data collection, and process and administrative forms will be identified by a coded unique identifier to maintain participant confidentiality. Access to collected data will initially be limited to fieldworkers at the point of data collection, and to the study statistician and investigators during analysis. Data to be collected is considered non-sensitive and does not include identifying participant information. Due to the nature of the mandate of the NMIMR, the institute is registered as a data controller with the Ghana National Data Protection Commission and abides by the provision of the Data Protection Act, 2012 (Act 843). Physical data is kept in locked cabinets with access restricted to only the investigators. The IT department provides the facility and secured server where each research fellow can securely store data. Moreover, the IRB requires a yearly report of the study, and may request to evaluate the data at any time. In addition to this, the ethical standards and guidelines of Horizon2020 and the Directive 95/46/EC on Data Protection and Privacy will be rigorously applied, throughout the study. Technical data-protection procedures and privacy/confidentiality measures will be implemented in the project, in compliance with national and European Union (EU) legal framework. Standard operating procedures will be developed to serve as guidelines and training for all study investigators, technicians and field personnel. All individuals who will participate in the proposed research will be required to sign the appropriate SOPs as evidence of training and understanding of the use of participants in biomedical research, and protection of personal data, and the principles for the design, conduct, monitoring, and reporting of clinical research (ICH E6 GCP).

#### Informed consent

This study will be embedded within the activities of the NTDP, which continues to deliver treatment to all endemic communities in the study areas. Written informed consent will be obtained from adults and parents or guardians of children, before enrolment (Additional file 2). Written informed consent will also be sought from individuals included in the qualitative evaluations, including FGDs and in-depth interviews. Participants of FGDs and interviews will be provided the options not to be quoted in any reporting of findings. Written assent to participate in the cross-sectional and longitudinal studies will be obtained from children aged 12 years and above. Risks and benefits of participating in the study will be presented during community meetings and any issues arising discussed. The risk of participating in the trial is very low. The study drugs, IVM and ALB, are safe and severe adverse events are not expected [1, 50]. However, side effects of treatment will be systematically monitored, through the system established by the NTDP. Health-care workers will remain in or near the villages for 48 h after drug administration. People who received treatment will be informed that fever and malaise are potential side effects of the two drugs and antipyretics will be given to those who complain of discomfort. In the unlikely situation of events occurring, these will be reported to the study site investigator and the principal investigator (PI) will inform the Ethics Committees. Collection of blood samples is a routine laboratory procedure. Trained phlebotomists will be used in all blood collection procedures. The potential adverse effects that may result from the study participants are listed below, with information on how to minimise them.

Potential adverse effects, discomfort or risks:

- Embarrassment in being selected for parasitological surveys
- Difficulty in collecting blood
- Lack of adherence with treatment
- Side effects from treatment

Steps to be taken to minimise adverse effects, discomfort or risks:

- The aims and objectives of the study will be explained to the study participants prior to recruitment. Study participants are free to leave the study at any time. All data will be anonymised, and no personal information will be traceable to the consenting participants
- Only trained phlebotomists will be used for blood collection. Best practices for blood collection, including calming the participant, will be employed
- Treatments will be directly observed by the CDDs, but will not be forced. This will also serve for monitoring compliance to MDA and monitoring side effects
- Adverse effects from IVM are minor, including itching, rashes, headache, nausea, vomiting, diarrhea, weakness, drowsiness. All participants will receive information to help identify and promptly report any adverse effects for management. Field personnel will also receive training in monitoring and managing side effects

#### Trial oversight

Ivermectin (400 μg/kg) and albendazole (400 mg), the drugs to be used in this study, are safe and used in the yearly mass treatment for the control of the disease. These drugs have already been delivered to millions of individuals each year as part of the GPELF, and over five billion doses have been distributed so far. Further, the 2015 Nobel Prize in Physiology or Medicine was awarded with one half jointly to Dr. William C. Campbell and Professor Satoshi Ōmura for their discoveries of avermectin (ivermectin), a therapy highly effective against infections caused by roundworm parasites in both animals and humans. As such, no data safety and monitoring board will be established since the interventions are safe. Instead, the NMIMR IRB will monitor data for quality and completeness. The NMIMR IRB and GHS-ERC will also review an interim analysis of the data on a yearly basis. The NMIMR IRB and GHS-ERC may also monitor the study at any time.

A project management team will be established, ensuring the quality of the data collected and results. The aim of the project management team will be to maintain the project on schedule, on budget and in accordance with local and international ethical principles and data-protection procedures. The project coordinator will work together with a clinical trials coordinator, with the tasks of overall project management, financial management, study team meetings and formal reporting. Financial management will be taken care of by the Office of Research Support, working closely and reporting directly to the coordinator. The responsibilities of the project coordinator will include the preparation and delivery of progress and final reports, as required by the funding agency, as well as any additional reporting that might be requested. The project coordinator will provide a project manual that will include information about project procedures, reporting expenses and actions and templates for reporting. This manual will be presented in the first study team meeting. The project coordinator will also manage the study team meetings. There will be seven study team meetings. In addition to the kick-off meeting at the beginning of the grant, there will two meetings per financial year. The project coordinator will take care of filing the project documentation (reports, minutes, plans, etc.) and will also provide and host a platform for sharing documents and storing documentation. The deliverables will be in the form of technical and financial reports.

The project management team will comprise the following:

- The study PI, coordinator
- A clinical trials coordinator
- A social scientist and epidemiologist
- A statistician and epidemiologist
- An administrative manager
- NMIMR IRB administrator
- NTD programme manager and project advisor
- NMIMR project advisors

Other individuals may also be invited to be part of the management team or to attend the project meetings.

### Dissemination

Besides the publication of results in peer-reviewed journals and presentations at international and national meetings, results will be disseminated in the study communities. The LF Control Programme uses community volunteers for education and drug administration. This approach will be intensified. After the study, community durbars will be organised to present the results of the study and educate members on the importance of control activities. Education will be reinforced based on locally generated data. The input of a cultural epidemiologist will be particularly relevant in this case, and one of the study investigators has been involved in developing programs that address social and cultural challenges to diseases control. The outcomes of the study will be communicated in local languages in order for the communities to understand the information being provided. The local media plays a significant role in the dissemination of information, and will be used in engaging the communities.

## Discussion

The persistent transmission of LF, following years of treatment, represents an important challenge to the LF-elimination programme in Ghana [8], and necessitates the need for additional, alternative strategies in order to achieve the elimination goals. In this study, it is assumed that all community members will actively take part in the MDA programme, in order to achieve high therapeutic coverage. While the selection of study participants is entirely voluntary, it is hoped that many non-compliant individuals will enrol in the study and actively participate in MDA programmes. Poor compliance has been reported in one hotspot district in Ghana [51], and our observations from previous studies in the districts suggest that many infected individuals are generally non-compliant with treatment [9]. An important reason given for non-compliance is the lack of information or inadequate information provided prior to the surveys and treatment [52]. Studies in one hotspot district have also reported low coverage and inaccurate data following MDA [22]. As such, the selection of study subjects will rely on intensive public education activities, informing the study communities on the importance of participating in epidemiological surveys and taking part in MDA. With effective compliance, twice-yearly treatment in high-endemic areas may be considered in reducing the duration of MDA to achieve interruption of transmission [8]. As such, extensive social mobilisation activities will be undertaken as part of the study.

The hotspot districts having received over 15 years of treatment [8] and the LF prevalence in study communities may be low, and thus this represents a challenge to assessing the impact of the intervention. Given sufficient budgetary allowance, the ideal would have been to screen many communities, out of which the study communities would be selected. However, this is not possible within the funding available for the study, and it is assumed that moderate infection prevalence will be obtained in order to evaluate the impact of the intervention.

At the end of this study, it is expected that firm recommendations of the twice-yearly treatment with IVM + ALB on speeding the elimination of LF as a public health problem and achieving the aims of the GPELF, will be made. The impact of twice-yearly versus yearly community treatment in reducing parasitological and entomological LF transmission indices will be determined, and the cost-effectiveness and community acceptability of twice-yearly treatment in reducing LF transmission will also be determined.

### Trial status

This trial is currently recruiting participants in study communities for parasitological assessments. Recruitment started on 19 May 2017 and is on-going at the time of submission of this manuscript.

## Additional files

Additional file 1: SPIRIT Checklist. (DOC 119 kb)

Additional file 2: Informed Consent Material. (DOCX 219 kb)

## Abbreviations

ALB: Albendazole
CDD: Community drug distributors
CFA: Circulating filarial antigen
DEC: Diethylcarbamazine
DOT: Directly Observed Treatment
EDCTP: European and Developing Countries Clinical Trials Partnership
ERC: Ethics Review Committee
EU: European Union
FGD: Focus group discussion
FTS: Filariasis test strip
FWA: Federal Wide Assurance
GCP: Good Clinical Practice
GHS: Ghana Health Service
GMI: Geometric mean intensity
GPELF: Global Programme for the Elimination of Lymphatic Filariasis
ICT: Immunochromatographic test
IRB: Institutional Review Board
IVM: Ivermectin
L3: Third-stage larvae of *W. bancrofti*
LF: Lymphatic filariasis
MDA: Mass drug administration
mf: Microfilaria
NMIMR: Noguchi Memorial Institute for Medical Research
NTDP: Neglected Tropical Diseases Program
PCR: Polymerase chain reaction
PSC: Pyrethrum spray collection
SAE: Serious adverse event
STH: Soil-transmitted helminthes
WHO: World Health Organisation
