# Supplementary material for: Community-based trial of annual versus biannual single-dose ivermectin plus albendazole against Wuchereria bancrofti infection in human and mosquito populations: study protocol for a cluster randomised controlled trial
Source: Trials. 2017 Oct 2;18:448. doi: 10.1186/s13063-017-2196-9 (PMC5625710; doi:10.1186/s13063-017-2196-9)
Supplement: Supplementary file 2 — Informed Consent Material. (DOCX 219 kb) [file 13063_2017_2196_MOESM2_ESM.docx]

**Participant information document**.

We are members of the Noguchi Memorial Institute for Medical Research here in Ghana, working together with the Ghana Health Service.

We want to do research on a disease called elephantiasis, which means that we want to learn more about elephantiasis, and how to control it.

I am going to give you information about this research and invite you and all other members of your household who are at least 5 years old to be part of this research. You do not have to decide today whether or not you or your child/ward will participate in the research.

Please interrupt me as soon as I say something you do not understand and ask me to explain. If you have questions later, you can ask a member of our team or call the people whose phone number we are giving you in this document. Before you decide, you can talk to anyone you feel comfortable with about the research.

**Why are we doing this research?**

Elephantiasis is a disease caused by tiny worms that live inside people. People can get these worms when they are bitten by mosquitoes. In worst cases, elephantiasis results in big legs in men and women, and swelling of the private part in men. Elephantiasis is very common in Ghana.


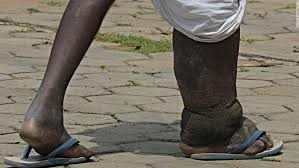


In the control of elephantiasis, the Ghana Health Service treats all members of communities where the disease is present. In this district, treatment has been going on since the year 2000. It is known that after 5-6 years of treatment, the disease can be eliminated. However, in this district there has been 15 years of treatment without success. In our research we want to find out whether treating people two times (June and December) in the year instead of the current one treatment (June only) per year will be better and faster at eliminating the disease.

The drugs given for the treatment by the GHS may also have some effects on other worms which can be found in your stomach, and therefore we can be able to assess worm infection as well, and the impact of the treatment on these.

**Why are we asking you to participate in this research?**

To do this research, we need people willing to be examined for worms in their blood and stool and for symptoms of elephantiasis and to answer questions that I will tell you more about later.

Our study will be undertaken in 18 different communities in the Ahanta and Axim districts. We are not able to enrol everyone in your community. We will examine 80 people, in each community, in order to assess the level of the disease, after which the communities will be grouped into two. One group will receive only one treatment in the year (June), and the other group will receive two treatments in the year (June and December). After treatment, all communities will be evaluated at 12, 24, and 30 months. Some participants will also be followed at 3, 6, 8 and 12 months to determine the impact of treatment on the worm.

**Who can participate in this research?**

People who are at least 5 years old and have lived in this community for at least one year can participate. Study participants should also be willing to agree to take part in the study, and donate a small amount of blood and stool.

People who feel sick or who cannot come out of their beds cannot participate.

It is your decision to participate in this research!

It is your choice whether to participate or not and to agree to participation of your child/ward or not. However you decide, this will not change the services you receive at health posts, health centers or hospitals when you or a member of your family are sick.

We will ask adults who agree to participate to confirm this by signature or thumbprint.

Children and adolescents younger than 18 years can only participate if a parent or guardian agrees and confirms this by signature or thumbprint and they themselves want to participate.

We will ask adolescents who are at least 12 years old to confirm their wish to participate via their signature.

**What will happen during the research?**

You will be examined by our team members at a private place at times that you agree to.

During the day, we will take a little bit of blood, about 10 drops of blood or less than half a tea spoon, from one of your fingers as shown in these photos.

| 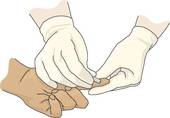 | 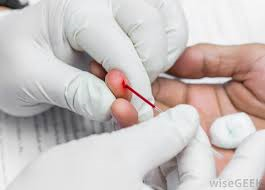 |
| --- | --- |

At that time we will also ask you about where you have lived before, your work and schooling, what you know about elephantiasis and how to protect yourselves from mosquito bites and which ways of protecting yourself and your family members you use. This will take about 10-15 minutes.

We will test the blood for signs that you have the worms that cause elephantiasis.

We will also give you a small container for you to provide us with a small amount of stool. All the stool samples will be collected the following day in the morning. They will also be analysed for other worms.

During the night, the worms that cause elephantiasis are going into the blood. Therefore, if we find signs of elephantiasis in the blood collected during the day, we will also set a date with you to come to your house again to collect about half a tea spoon of blood from your arm (as shown in the picture below) between 9pm and midnight so we can do more tests for the worms.

[
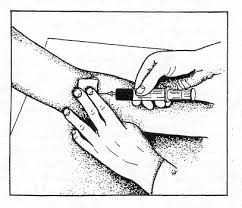
](https://www.google.com.gh/imgres?imgurl=http://www.sweethaven02.com/MDSeries/MD0853/framew26.gif&imgrefurl=http://www.waybuilder.net/free-ed/Courses/06%20MedHealth/MD%20Series/MD0853/Hematology.asp?iNum=0301&docid=w5Yhg72AHalDPM&tbnid=ZNmkSRjNZom_1M:&w=407&h=350&bih=855&biw=1200&ved=0ahUKEwi6ubSo8IHPAhUHWRoKHf98BH0QMwhmKCswKw&iact=mrc&uact=8)

We will also collect mosquitoes from your house, to see if the mosquitoes have the elephantiasis worms. For this collection, two of us will lay some white bedsheets in a bedroom that you agree for us to enter. After that, we will spray the common mosquito spray that you can buy in the market and close the door for about 15 minutes. The spray will kill the mosquitoes which will fall on the bedsheets. We will then remove the bedsheets and pick the dead mosquitoes (as shown in the picture below).


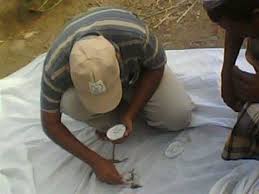


Many mosquitoes enter the house overnight. Therefore we have to catch them between 5 and 8 in the morning. It will take us around 30 minutes. Please tell us which time will be best for you.

If you agree to let us collect mosquitoes in your house, you need to do the following:

- When you wake up in the morning, please close all windows and doors in the room where we should collect the mosquitoes.
- Please remove all animals from that room.
- Please remove or cover all food items.
- We will open the windows and doors of the room before we leave so that fresh air can enter it. Please do not enter the room for 15 minutes after we have left.

**What are the discomforts and pain that you may experience and how will we make sure that you get care if you need it?**

You may experience some discomfort from the needle prick during the blood taking process. This procedure is the same as is usually done in a hospital laboratory.

We will also require 10-15 minutes of your time to ask you some questions about the disease.

Should you experience any discomfort from the drugs, please contact the community drug distributor/nurse who will arrange for your care at the health post/center or hospital. After treatment, a nurse will be available in your community for two days. Please report all problems with the drug to him/her. Any discomfort after two days should be reported to the community drug distributor who will direct you to the community nurse or district health management team. You can also contact us directly, and we will send the nurse directly to you. Any care provided to you at the health post/center/hospital as a result of your participation in this study will be at no cost to you (that is, we will pay for all the cost).

**Will you have any benefits from participating in this research?**

You will learn whether you have worms that cause elephantiasis and other diseases. If you have them we will provide you with drugs to take.

We will also give you advice on how best to protect yourselves from mosquito bites.

You will also help us to understand whether elephantiasis can be eliminated by treating once a year or twice a year, and the Ghana Health Service will decide what to do about it.

**What compensation will you get for the time you spend on this research?**

For the time lost for working when we take blood, ask you questions or examine you during the day, you will receive 7 GHS.

**Will you and your communities be informed about the results of our research?**

We will inform you personally about the results of your tests and examination.

After the end of our research, there will be a meeting to brief the community about the outcome of our research. During the meeting we will inform the community whether there are many people with elephantiasis and other disease worms in this community or other communities in this district and what the Ghana Health Service plans to do about it.

**Who will know about your participation in this research and who will know the results of our research?**

We will not tell anybody outside our research team who is participating in this research. Others in your community may see that you are participating when you talk to members of our team.

If you agree to participate, we will replace your name with a number. The samples we collect, the worms we find and your answers to our questions will be identified only with that number. No one, outside the study investigators, will be able to link you to the answers, samples and worms. Information that is entered on paper will be under lock and key, and information entered on a computer will be protected and only accessible to the study investigators.

We will share the data and results from our research with other people in Ghana and other countries doing research. Nobody with whom we share this will know who participated in the research.

**What will we do with left over blood or worms?**

If any blood or worms are still good for further research after we have completed the research we are telling you about here, we will keep them for up to 10 years. They will be used for other research that will allow us to learn more about how best to diagnose or fight diseases caused by worms. Any information (such as your name) that can link you to the blood will be removed. If you do not want us to keep your blood please let us know. Any blood that does not contain the worms or is not good, will be discarded.

Should we find that the blood or worms could be useful for other types of research, we will ask the Ethics Committees which have approved this research for permission. Ethics Committees are groups of people who review plans for research to ensure that the research is well done and does not cause avoidable harm to the participants.

**You can refuse to participate!**

As we already told you, you or your child/ward do not have to take part in this research if you do not wish to.

If you agree and later change your mind, you just have to tell us. In that case, we will remove all your information that we have already collected from the study, and we will not contact you or your child/ward further. Your sample would also be discarded.

Should we learn something during our research that might make you change your mind about participating, we will inform you about this and ask you whether you want to continue to participate.

**Who can you contact if you have more questions?**

If you have any questions you may ask them now or later, even after the study has started. You can call the investigators on the numbers provided above.

This research has been reviewed and approved by the Ethics Committees of the Ghana Health Service and the Noguchi Memorial Institute for Medical Research. You can also contact them on the numbers provided.

We will give you a copy of this information sheet. If you agree to participate or for your child/ward to participate, we will give you one copy of the signed Volunteer Agreement to keep.

**Volunteer Agreement / Consent**

I confirm that I have read the Participant Information Document explaining this research project on elephantiasis or that it has been read to me. I have been given a chance to ask questions until I feel that all of my questions have been answered. I know that nobody can force me to agree to be part of this research and that I can change my mind at any time.

I agree to give samples, allow mosquitoes to be collected from my room and answer questions related to the disease. Tick which ever applies and cross out those that do not apply.

Blood [ ] Stool [ ] Mosquitoes [ ] Questionnaire [ ]

Storage of left over Blood? [ ]

|  |  | |  |
| --- | --- | --- | --- |
| Date | Name of volunteer | | Signature/mark/thumbprint of volunteer |
| District: | | Village: | |

**WITNESS STATEMENT**

*If volunteers cannot read the form themselves, a witness must confirm:*

I was present while the information document was read and explained. All questions were answered and the volunteer has agreed to take part in the research.

|  |  |  |
| --- | --- | --- |
| Date | Name of witness | Signature of witness |

**STUDY TEAM MEMBER STATEMENT AND SIGNATURE**

I certify that the information document was read by/to the volunteer, that I answered all questions and that the volunteer agreed to participate.

|  |  |  |
| --- | --- | --- |
| Date | Name of survey team member who informed the participant | Signature of survey team member |

**PARENTAL / GUARDIAN CONSENT for participation of minors 5-17 years**

I confirm that I have read the Participant Information Sheet explaining this research project on elephantiasis or that it has been read to me. I have been given a chance to ask questions until I feel that all of my questions have been answered. I know that nobody can force me to agree that my child/ward participates in this research and that I can change my mind at any time.

I agree that my child/ward can give samples and answer questions related to the disease. Tick which ever applies, and cross out those that do not apply.

Blood [ ] Stool [ ] Questionnaire [ ] Storage of left over Blood? [ ]

| Minor's name |  | | |
| --- | --- | --- | --- |
|  |  | |  |
| Date | Name of parent/guardian | | Signature/mark/thumbprint of parent/guardian |
| District: | | Village: | |

**WITNESS STATEMENT**

*If parent/guardian cannot read the form themselves, a witness must confirm:*

I was present while the information document was read and explained. All questions were answered and the parent/guardian has agreed to let the child/ward take part in the research.

|  |  |  |
| --- | --- | --- |
| Date | Name of witness | Signature of witness |

**STUDY TEAM MEMBER STATEMENT AND SIGNATURE**

I certify that the information document was read by/to the parent/guardian, that I answered all questions and that the parent/guardian agreed to participation of their child/ward.

|  |  |  |
| --- | --- | --- |
| Date | Name of survey team member who informed the participant | Signature of survey team member |

**Volunteer Agreement / ASSENT – for adolescents 12 to 17 years**

I confirm that I have read the Participant Information Document explaining this research project on elephantiasis or that it has been read to me. I have been given a chance to ask questions until I feel that all of my questions have been answered. I know that nobody can force me to agree to be part of this research and that I can change my mind at any time.

I agree to give samples and answer questions related to the disease. Tick which ever applies.

Blood [ ] Stool [ ] Questionnaire [ ] Storage of left over Blood? [ ]

|  |  | |  |
| --- | --- | --- | --- |
| Date | Name of adolescent volunteer | | Signature of volunteer |
| District: | | Village: | |

**WITNESS STATEMENT**

*If the volunteer is an adolescent, a witness must confirm:*

I was present while the information document was read and explained. All questions were answered and the volunteer has agreed to take part in the research.

|  |  |  |
| --- | --- | --- |
| Date | Name of witness | Signature of witness |

**STUDY TEAM MEMBER STATEMENT AND SIGNATURE**

I certify that the information document was read by/to the volunteer, that I answered all questions and that the volunteer agreed to participate.

|  |  |  |
| --- | --- | --- |
| Date | Name of survey team member who informed the participant | Signature of survey team member |
